# Melatonin in Antinociception: Its Therapeutic Applications

**DOI:** 10.2174/157015912800604489

**Published:** 2012-06

**Authors:** Venkatramanujam Srinivasan, Edward C Lauterbach, Khek Yu Ho, Dario Acuña-Castroviejo, Rahimah Zakaria, Amnon Brzezinski

**Affiliations:** 1 Sri Sathya Sai Medical Educational and Research Foundation, Medical Sciences Research Study Center, Prasanthi Nilayam, 40 Kovai Thirunagar, Coimbatore-641014, Tamilnadu, India; 2 Department of Psychiatry and Internal Medicine (Neurology Section), Mercer University School of Medicine, Macon GA31201, USA; 3 Department of Medicine, National University Hospital, National University of Singapore Lowerkent Bridge Road, Singapore; 4 Instituto def Biotecnología, Centro de Investigaicón Biomédica, Parque Tecnológico de Ciencias de la Salud, Universidad de Granada, Avda del Conocimiento, 18100-Armilla, Granada, Spain; 5 Department of Physiology, School of Medical Sciences, Universiti Sains Malaysia, 16150, Kubang Kerian, Kelantan, Malaysia; 6 Department of Obstetrics and Gynecology, Hadassah Medical Center, The Hebrew University, Jerusalem, Israel

**Keywords:** Pain, nociception, analgesia, melatonin, inflammatory, neuropathic, fibromyalgia, cluster headache, migraine.

## Abstract

The intensity of pain sensation exhibits marked day and night variations. Since the intensity of pain perception is low during dark hours of the night when melatonin levels are high, this hormone has been implicated as one of the prime antinociceptive substances. A number of studies have examined the antinociceptive role of melatonin in acute, inflammatory and neuropathic pain animal models. It has been demonstrated that melatonin exerts antinociceptive actions by acting at both spinal cord and supraspinal levels. The mechanism of antinociceptive actions of melatonin involves opioid, benzodiazepine, α_1_- and α_2_-adrenergic, serotonergic and cholinergic receptors. Most importantly however, the involvement of MT_1_/MT_2_ melatonergic receptors in the spinal cord has been well documented as an antinociceptive mechanism in a number of animal models of pain perception. Exogenous melatonin has been used effectively in the management of pain in medical conditions such as fibromyalgia, irritable bowel syndrome and migraine and cluster headache. Melatonin has been tried during surgical operating conditions and has been shown to enhance both preoperative and post-operative analgesia. The present review discusses the available evidence indicating that melatonin, acting through MT_1_/MT_2_ melatonin receptors, plays an important role in the pathophysiological mechanism of pain.

## 1 INTRODUCTION

Pain is classically described as an unpleasant sensation. By definition “pain is an unpleasant sensory and emotional experience associated with actual or potential tissue damage” [1]. ***Clinical pain*** has been divided into two conditions, including ***inflammatory pain*** caused by inflammation and ***neuropathic pain*** caused by actual injury to the nervous system [2]. These types of pain are characterized by persistent pain hypersensitivity [2]. In addition to hyperalgesia, neuropathic pain also manifests as allodynia (pain occurring in response to normally innocuous stimuli). Multiple nociceptive mechanisms have been suggested to underlie neuropathic pain [3]. As this type of pain can persist for years, it is also associated with symptoms like anxiety, depression, and sleep-disturbances [4, 5]. On the other hand symptoms of pain can also constitute a presenting feature of major depressive disorder, suggesting pathophysiological similarities between clinical pain and certain psychiatric disorders, particularly major depression [6, 7]. The mechanism of pain perception is considered to be multifactorial involving many biochemical, humoral, neurophysiological and psychological factors [8]. Damage or inflammation of tissue releases a variety of inflammatory mediators such as prostaglandins (PGE_2_), leukotrienes, bradykinin, substance P and inflammatory cytokines like tumor-necrosis factor (TNF-α), ATP and adenosine. Each of these substances either directly activates nociceptors or releases local allogenic agents which sensitize nociceptors and enhance the neuronal excitability of pain transmission pathways [9]. The transmission of pain sensation involves nociceptors (the primary sensory nerve endings of Aδ and C fibers) that have their central processes in the dorsal horn of the spinal cord.

The dorsal horn of the spinal cord is the first site of synaptic transfer in the nociceptive pathway and it is the primary region where peripheral afferent signals are integrated and modulated. A number of neurotransmitter-receptor sites such as NMDA, AMPA, opioid (µ and δ), α_2_-adrenoceptors, and adenosine are located in the dorsal horn projection neurons. The dorsal horn region of the spinal cord is the region of central sensitization and this is mediated by neurotransmitters like glutamate, substance P and neurokinin which interact with the NMDA (N-methyl-D-aspartic acid) receptor system [10, 11]. In addition to this, the dorsal horn neuron is also subjected to the influences of interneurons of the dorsal horn itself and descending pathways that modulate pain perception [7, 12]. These spinal interneurons and descending neural tracts from the brain constitute endogenous pain modulatory systems that inhibit pain transmission signals. These pain modulatory systems are activated by opioid and GABAergic mechanisms located in and around the periaqueductal gray region (PAG). From the PAG, descending fibers project to the rostral ventrolateral medulla (RVM) and dorsolateral pontine structures that in turn send inhibitory projections to the spinal cord to induce antinociceptive effects. Inhibitory α_2_-adrenergic, δ, µ, and κ opioid and 5-HT_1A_ receptors are present in the post-synaptic dorsal horn neurons along with GABA-A/B receptors [7, 13]. Descending fibres synapse with primary afferent neurons, and secondary neurons or interneurons to stimulate them to release opioid peptides.

The management and control of pain sensation is a matter of intense study and of great clinical interest. Drugs like aspirin and non-steroidal anti-inflammatory drugs (NSAIDS) exert their antinociceptive effects by inhibiting cyclooxygenases (COX) [14]. But the side effects of aspirin and NSAIDS including gastrointestinal bleeding and ulceration make them unsafe for long-term clinical use. Other classes of drugs like morphine or the anti-inflammatory drugs that act through the NO-cyclic GMP pathway exert their antinociceptive actions by blocking the ascending transmission of pain sensation [15]. The side effects associated with morphine including sedation, respiratory depression and dependence complicate its use in controlling nociceptive mechanisms [16, 17]. In addition there are many other painful conditions like neurodegenerative pain and varieties of neuropathic pain that are not responsive to potent analgesics including opioids. Neuropathic pain is also a frequent manifestation of neurodegenerative diseases. Although it is less appreciated in Parkinson’s disease (PD), the prevalence of pain is nearly 40% in PD. Pain presentations in PD include several categories such as musculoskeletal pain, radicular or neuropathic pain, and primary central parkinsonian pain [18]. Neurodegenerative mitochondrial and cytoskeletal cellular mechanisms producing painful hyperactivity in primary afferent nociceptors have been suggested as possible causative factors contributing to hyperalgesia [19]. Tricyclic anti-depressants and gabapentin are often prescribed for the treatment of neuropathic pain with variable results of response [20-22], suggesting thereby the need for the development of novel analgesic drugs that can be used for effective treatment and management of inflammatory and neuropathic pain.

## 2 MELATONIN AND NOCICEPTION

Diurnal variations in the perception of pain have been described both in rodents and humans [23-26]. It has been reported that patients suffer less pain during the dark phase of the photoperiod and prolonged latencies in pain thresholds have been detected in healthy human subjects during night time. These observations were recorded by Laikin and his co-workers [26] and they attributed this phenomenon to high melatonin levels occurring at night and their possible analgesic effects. Based upon this initial observation a number of investigators have evaluated the antinociceptive effects of melatonin in animals by using a variety of experimental models and designs.

## 3 MELATONIN BIOSYNTHESIS

**Fig. (1):**
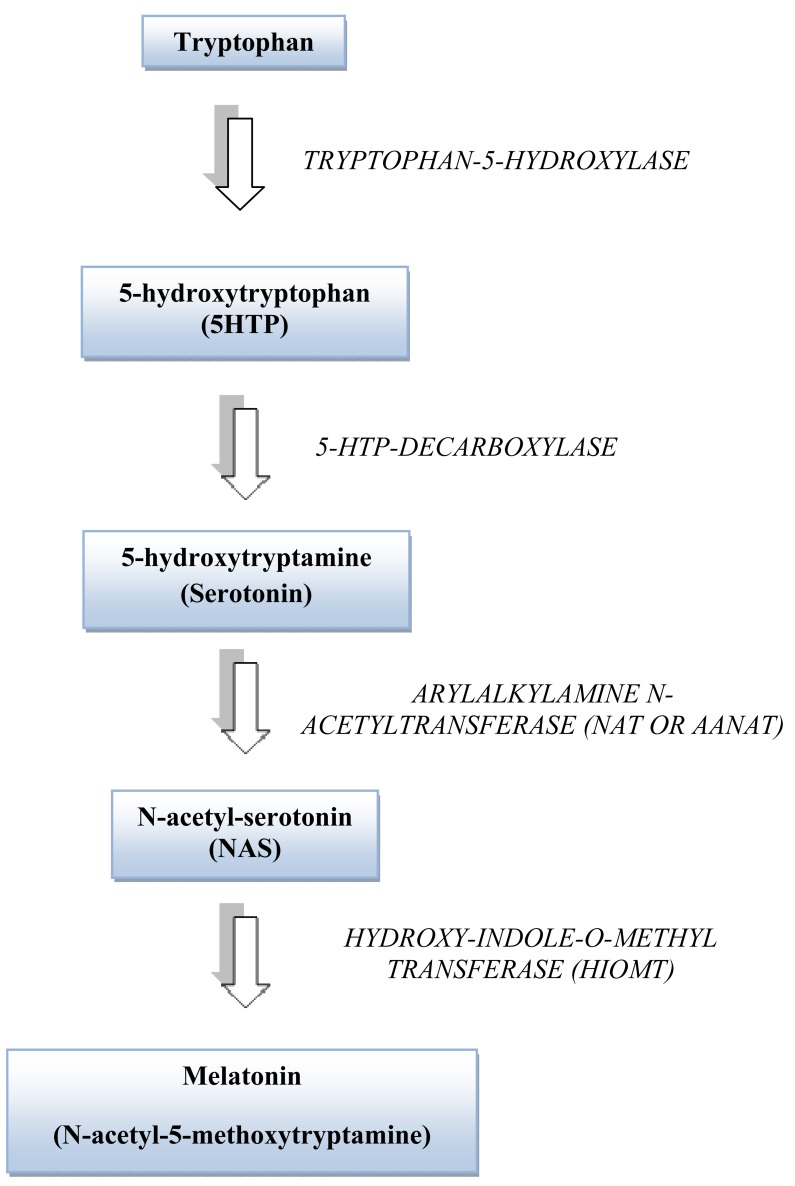
Melatonin Biosynthesis.

Melatonin (N-acetyl-5-methoxytryptamine) is a neurohormone produced mainly in the pineal gland of all vertebrates in a circadian fashion [27]. Tryptophan serves as the precursor for its biosynthesis and is converted into 5-hydroxytrptophan. Serotonin is then acetylated to form N-acetylserotonin by the enzyme arylalkylamine N-acetyltransferase (NAT) and then O-methylated by hydroxy-indole-O-methyltransferase (HIOMT) to form melatonin (Fig. **1**). Biosynthesis of melatonin in the pineal gland is regulated by the suprachiasmatic nucleus (SCN) of the hypothalamus and is synchronized to the environmental light-dark cycle [28, 29]. Special melanopsin containing ganglion cells [30] transmit this photoperiodic information to the SCN through the retino-hypothalamic tract [31]. Fibers from the SCN project to the superior cervical ganglion (SCG) through a circuitous route. Post-ganglionic fibers from SCG regulate pineal melatonin synthesis by releasing norepinephrine (NE) at pinealocyte receptor sites. NE released from the postganglionic sympathetic fibers interacts with β-adrenergic receptors as well as α_1_-adrenergic receptors of the pinealocyte membrane, activating the adenyl cyclase cyclic AMP pathway that in turn regulates the expression of NAT and other enzymes involved in melatonin biosynthesis [32]. Exposure to bright light during dark hours of the night suppresses melatonin production by degradation of NAT enzyme [33]. Once formed, melatonin is not stored in the pineal gland but diffuses into the blood [34]. But most of the melatonin from the pineal gland is also released simultaneously into the cerebrospinal fluid (CSF) in primates [35]. The concentration of melatonin in the CSF of the third ventricle is 20 to 30 times higher than that found in the blood [36].

Circulating melatonin is metabolized mainly in the liver where it is first hydroxylated in the C6 position by cytochrome P_450_ mono-oxygenases (isoenzymes CYP1A2, CYP1A1, and to a lesser extent CYP1B1) and thereafter conjugated with sulphate to be excreted as 6-sufatoxy-melatonin (aMT6S). In the brain a substantial amount of melatonin is metabolized to kynuramine derivatives [37]. This is of interest as antioxidant and anti-inflammatory properties of melatonin are shared by some of these metabolites, N^1^–acetyl-N^2^–formyl-5-methoxy kynuramine (AFMK) and also by N^2^-acetyl-5-methoxykynuramine (AMK) [38].

Melatonin is also synthesized in many other regions of the body like skin [39], gastrointestinal tract, lymphocytes [40] and thymus [41]. In these regions melatonin has either a paracrine or autocrine role. Among other functions, extrapineal melatonin has a modulatory role on inflammation, reducing hyperalgesia, thus contributing to pain control [42]. Melatonin is synthesized by a number of plants. Of these, St John’s Wort (*Hypericum* *perforatum*) is of medical importance and it has very high concentrations of melatonin [43, 44]. In a recent study it is reported that extracts of *Hypericum perforatum *(St John’s Wort or SJW), *Harpagophytum* *procumbens *(HPE)** and *grape* *seed* *proanthocyanidins* (GSPE) exerted significant antinociceptive effects in mice [45]. Nearly 60 commonly used Chinese medicinal herbs contain melatonin in concentrations ranging from 12 to 3771 ng/g [46]. It is an interesting fact that many of these melatonin containing herbs used in Chinese traditional medicine have age retarding effects and have been in use for treating diseases associated with increased generation of free radicals. Indeed the presence of melatonin in plants helps to protect them from oxidative damage and also from many environmental insults to which they have been subjected [47, 48]. Melatonin was observed to be elevated in alpine and Mediterranean plants exposed to strong UV radiation, a finding amenable to the interpretation that melatonin’s antioxidant properties can antagonize damage caused by light-induced oxidants [49]. Melatonin in plants serves as a good antioxidant nutrient. The high concentration of melatonin detected in seeds also provide anti-oxidative defence even in a dormant and more or less dry system when most of the other substances and enzymes are not able to provide their full capacity as antioxidants [50].

## 4 MELATONIN RECEPTORS 

Most of the physiological and pharmacological actions of melatonin are mediated through the activation of high affinity G-protein coupled receptors, namely MT_1_ and MT_2_ and belong to the seven-transmembrane receptor family [51-53]. Melatonin binding to nuclear receptors has been demonstrated [54]. Some of these binding sites were identified as belonging to retinoid orphan related receptors like RZRα and RZRβ, and they are present in the central and peripheral nervous systems [55]. Both MT_1_ and MT_2_ receptors have been identified in the ventral and dorsal horn of thoracic and lumbar regions of the spinal cord, specifically in lamina I-V and X of the spinal cord which are involved in the pain regulatory mechanisms [56]. Earlier studies have localized melatonin receptors in different areas of the brain like the thalamus, hypothalamus, spinal trigeminal tract and trigeminal nucleus [57, 58].

## 5 MELATONIN’S ANTINOCICEPTIVE ACTION: EVIDENCES FROM ANIMAL MODELS

Melatonin’s antinociceptive effects have been demonstrated in animals like mice and rats by using a number of experimental models of nociception and this has been brought out extensively in an earlier review [59]. A brief analysis of this is presented in this paper with the aim of understanding the clinical significance of the antinociceptive and beneficial effects of melatonin. Using the hotplate procedure, it was shown that a melatonin injection (20-40 mg/kg, i.p) exerted its maximal analgesic effect when given in the late afternoon. This analgesic effect was blunted by the administration of either the opiate antagonist naloxone or the central benzodiazepine antagonist flumazenil, showing thereby the involvement of central opioid or benzodiazepine (BZD) receptors [60]. Soon after, it was showed that β-endorphin, through µ-opioid receptors, is involved in melatonin-induced modulation of brain BZD receptors [61]. Dose-dependent antinociceptive effects of melatonin were evaluated by means of the hot-water tail flick test in a group of rats in which melatonin, injected (i.p) at three different doses (30, 60, 120 mg/kg), produced dose-dependent antinociception [62]. The antinociceptive effect began within 15 minutes after injection and reached a peak in 30 minutes, and with 60-120 mg/day doses, lasted for 100 minutes. Melatonin’s antinociceptive effect with 60 to 120 mg/kg (i.p) was antagonized by an i.c.v injection of naloxone within 10 minutes after injection and lasted for 45 minutes. As the i.c.v injection of naloxone blocked melatonin’s antinociceptive effect it was concluded that the CNS is the primary site for melatonin’s antinociceptive effect. In another study, it was found that melatonin potentiated the antinociceptive effects of deltorphin-1 a, δ-opioid agonist but not of the µ-opioid agonist endomorphin-1. In this study melatonin was injected either i.p (1, 5, 25 mg/kg) or i.c.v (0.25, 0.5, 1 mg/kg), which produced significant tail withdrawal latencies, thereby confirming antinociceptive effects of melatonin [63]. A summary of the antinociceptive effects of melatonin in a variety of animal models is presented in Table **1**.

## 6 INFLAMMATORY TYPE OF PAIN AND MELATONIN’S ANTINOCICEPTIVE EFFECTS

Lipopolysaccharide (LPS) injection into the hind paws of mice (5 µg in 50 µl saline) significantly decreased the nociceptive threshold in the hot-plate and tail-flick tests 6 and 10 hrs after injection. Prior injection of melatonin (5 or 10 mg/kg) significantly inhibited LPS-induced hyperalgesia at both time intervals. A noted finding of this study was that melatonin‘s effect on LPS-induced hyperalgesia was not reversed by the opiate antagonist naltrexone (4 mg/kg) [64]. In carrageenan-induced inflammation in rats, melatonin injection (0.5 and 1.0 mg/kg, i.p) increased nociceptive thresholds [65]. Melatonin also increased the antinociceptive effects of indomethacin and, further, reduced carrageenan-induced paw oedema. Melatonin has been found to reduce the release of prostaglandins and recruitment of polymorphonuclear leukocytes at the inflammatory site in carrageenan treated rats [66]. In a recent animal model study of inflammatory pain, O_2_^-^ (super oxide anion) injected into the right paw of rats resulted in the generation of peroxynitrite anion (presumably by interacting with endogenous nitric oxide (NO) and produced hyperalgesia [42]. Prior injection of melatonin in 25 to 100 mg/kg doses attenuated the hyperalgesic responses to O_2_^-^. Melatonin has been suggested to inhibit inflammation and tissue damage by affecting COX-2 and inhibitory nitric oxide synthase expression (iNOS) [42]. Not only melatonin, but melatonin analogues such as the pyrrolol [1, 2α] indole derivatives 3, 5, 12, 14 also exerted significant anti-inflammatory and analgesic effects in the carrageenan-induced paw oedema study [67]. Melatonin also inhibited capsaicin-induced hyperalgesia and naloxone pre-treatment completely reversed melatonin’s antinociceptive effects [68]. To evaluate the role of the spinal melatonin system in impeding the initiation or generation of capsaicin-induced secondary mechanical allodynia and hyperalgesia, melatonin or its antagonist 4-phenyl-2-propionamido-tetraline (4-P-PDOT) was administered intrathecally 30 minutes prior to capsaicin injection [69]. Intrathecal pre-administration of melatonin or its agonist significantly decreased or completely blocked the enhanced responses to von Frey filament stimulation following the injection of capsaicin. Melatonin also limited the intensity and duration of the secondary mechanical allodynia, and hyperalgesia, to mechanical stimulation following capsaicin injection. From this study it is concluded that the spinal melatonin system has the ability to impede the initiation and limit the development of “central sensitization” induced by capsaicin injection [69]. In the same study the effect of 6-chloromelatonin on capsaicin-induced mechanical allodynia and hyperalgesia was evaluated. The increase in paw withdrawal frequency induced by capsaicin was significantly reduced by intrathecal injection of the melatonin agonist 6-chloromelatonin. This antinociceptive effect of 6-chloromelatonin was blocked by intrathecal administration of the MT_2_ receptor selective antagonist 4-P-PDOT, suggesting thereby the involvement of spinal MT_2_ receptors in nociceptive transmission mechanisms. 

The formalin test is used as a model for assessing nociceptive behaviour. Subcutaneous injection of 5% formalin produces a licking response of the injected hindpaw in a biphasic pattern, involving rapid and brief withdrawal of the affected paw. The two phases of the licking/flinching response are phase 1 (0-9 minutes) and phase-2 (10-60 minutes). Melatonin administration decreased the licking/flinching responses in both phases [70-72]. In a recent study on formalin injection, intrathecal melatonin reduced the flinching response during phase-1 and phase-2, thereby attenuating both the facilitated state and acute pain evoked by formalin injection [73]. The antinociceptive effects of melatonin in some studies [74-76] also are presented in Table **1**.

## 7 NEUROPATHIC PAIN AND ANTINOCICEPTIVE ROLE OF MELATONIN

Neuropathic pain due to nerve injury is associated with thermal hyperalgesia and mechanical allodynia. Partial tight ligation of the sciatic nerve in rats is a widely employed model, which produces spontaneous pain, allodynia, and hyperalgesia analogous to the clinical conditions of neuropathic pain [77-79]. In a study conducted in mice, ligation of the sciatic nerve produced pain-like behaviour characterized by mechanical allodynia and thermal hyperalgesia [80]. Injection of melatonin at high doses (120 mg/kg, i.p) and 0.1 nmol i.c.v, reduced paw withdrawal latencies in response to radiant heat stimulation of the injured hindpaw. Concomitant administrations of both L-arginine (200 mg/kg, i.p; 80 µg, i.c.v) and naloxone (1 mg/kg, i.p; 10µg, i.c.v) reduced the effect of melatonin on thermal hyperalgesia. The findings of this study show that melatonin’s effect on thermal hyperalgesia is mediated partially through the L-arginine-NO-pathway [80]. In this study melatonin even at high doses (either through i.p or i.c.v) did not have any effect on mechanical allodynia. However in another study of neuropathic pain, using the L5/L6 spinal nerve ligation rat model, intrathecal (3-100 µg) or oral (37.5-300 mg/kg) administration of melatonin decreased tactile allodynia induced by spinal nerve ligation [81]. 

There is evidence that melatonin interacts with glutamatergic systems involving the NMDA receptor. In the study just mentioned, the concomitant administration of both melatonin (30mg/kg) and dextromorphin 15 mg/kg effectively reversed both thermal hyperalgesia and mechanical allodynia induced by ligation of spinal nerves, thereby suggesting the involvement of both melatonin MT_2_ receptors and NMDA receptors in nociceptive mechanisms [81]. This antiallodynic effect of melatonin was diminished by intrathecal administration of luzindole (1-100 µg), a common MT_1_/MT_2_ receptor antagonist, and by oral (0.01 to 1.0 mg/kg) or intrathecal (0.1 to 10 µg) 4P-PDOT, a selective MT_2_ receptor antagonist, suggesting the involvement of melatonin MT_2_ receptors in the antiallodynic effects of melatonin [81]. Dextromethomorphin is an NMDA receptor (NMDAR) antagonist and it is used for treating patients with neuropathic pain such as complex regional pain syndrome and painful diabetic neuropathy. Increased NMDAR activity contributes to central sensitization in certain types of neuropathic pain. Clinically used NMDAR antagonists such as ketamine and dextromethomorphin are effective in treating neuropathic pain [82].

## 8 ANTINOCICEPTIVE EFFECTS OF MELATONIN CONTAINING PLANT - ST JOHN’S WORT

High concentrations of melatonin have been reported in several medicinal plants including *Hypericum perforatum *(St John’s Wort or SJW) [43]. The extract from SJW has been shown to possess clinical efficacy in treating moderate depression [83]. *In vitro* studies have shown that SJW extracts act by blocking the reuptake of serotonin and noradrenaline like tricyclic antidepressants [84-86]. By employing the formalin and tail-flick tests of nociception, the antinociceptive effect of SJW was assessed in mice. SJW at doses of 100-1000 mg/kg significantly reduced the licking and biting time both in phase 1 and phase 2 in a dose-dependent manner, showing thereby its antinociceptive effect. As oral administration of SJW significantly inhibited the formalin-evoked increase of nitric oxide (NO) content in the spinal cord, it was concluded that the antinociceptive action of SJW is related to suppression of the spinal NO pathway [45]. Similar effects of melatonin are possible and cannot be excluded, given the high concentrations of melatonin in SJW.

## 9 MELATONIN’S ANTINOCICEPTIVE EFFECTS: EVIDENCES FROM CLINICAL STUDIES 

Chronic pain is encountered in a number of clinical conditions such as fibromyalgia, pelvic pain conditions, irritable bowel syndrome, tension and cluster headaches, migraine, etc. [87]. Similarly a number of psychiatric conditions are associated with abnormal pain sensations. Pain thresholds in patients with post-traumatic stress disorder (PTSD) are significantly higher than controls or in patients with other conditions [88, 89]. Patients with depressive disorders suffer from painful symptoms like neck and back pain and stomach pain [90], and painful symptoms in general are common in major depressive disorder [6]. Conversely, chronic pain is suggested as a major risk factor for the development of major depressive disorder [91]. 

The link between pain and depressive disorders has been well studied and it is suggested that a common pathophysiology may perhaps underlie these disorders [92, 93]. Serotonergic cell bodies located in the raphe nucleus and noradrenergic neurons located in the locus coeruleus project to various parts of the brain involved in the control of mood. The locus coeruleus and dorsal part of the raphe nucleus also project down to the spinal cord [94]. These descending pathways inhibit the transmission of pain sensations from sensory neurons of the dorsal horn of the spinal cord. Inhibitory α_2_-adrenergic and 5-HT_2A_ receptors are also present on dorsal horn neurons [95]. A dysfunction of the descending neuronal pathways from the raphe nucleus and locus coeruleus can result in hypersensitivity of pain transmission systems, resulting in hyperalgesia and allodynia [7]. As pain and depression share common neuronal systems, drugs acting on serotonergic and noradrenergic systems will be beneficial in treating not only depressive disorders but also in treating chronic clinical disorders of pain [7]. Perhaps this is best illustrated in conditions of neuropathic pain, where treatment with potent analgesics including opioids has been ineffective [22, 96]. Hence, tricyclic antidepressants are often prescribed in the clinical management of neuropathic pain [21, 22]. 

Simultaneous effectiveness against pain and depression is not restricted to conventional antidepressants, and melatonergic antidepressants offer additional efficacy. Agomelatine, a novel melatonergic agonist, has been shown to be effective in treating patients with major depressive disorders and has been used successfully in a number of clinical studies conducted in Europe and the USA [97-100]. Use of agomelatine may be effective in the treatment of major depressive disorders associated with painful conditions and it may offer the potential for effective management and control of neuropathic pain.

### a Melatonin use in Fibromyalgia

Fibromyalgia is a clinical condition in which widespread debilitating musculoskeletal pain, hyperalgesia, allodynia and stiffness of the body are found [101]. These patients suffer from sleep disturbances, depressive and anxiety symptoms, and impairment of memory and cognitive functions collectively known as “fibrofog” [102-104]. The pathophysiology of fibromyalgia is said to be due to abnormalities of the central pain processing mechanisms that cause central pain sensitization [105-108]. Although few studies have carried out melatonin measurements in patients with fibromyalgia, a single study of 8 fibromyalgia patients showed significantly lower plasma and urinary melatonin (between 23:00 hrs and 07:00 hrs) levels when compared to controls [109]. Treatment of fibromyalgia patients with melatonin has been carried out in two studies. In the first open label study carried out on 21 female patients, melatonin was administered in doses of 3 mg orally for 4 weeks 30 minutes before bed time [110]. Improvements with regard to pain, fatigue and depressive symptoms were noted in these patients with melatonin therapy. In the second double-blind placebo controlled study in 101 fibromyalgia patients, different doses of melatonin were used either alone or in combination with fluoxetine [111]. Patients were divided into four groups: group A (24) patients were treated with fluoxetine alone; patients of group B (27) were treated with melatonin 5 mg/day, group C (27) patients were treated with fluoxetine (20 mg/day) and melatonin 3 mg/day, group D patients (23) were treated with fluoxetine (20 mg/day) and melatonin 5 mg/day. Fluoxetine was given daily in the morning and melatonin was given each evening for 8 weeks. Treatment with fluoxetine alone improved symptoms including fatigue, anxiety, depression and morning stiffness. Treatment with melatonin alone caused significant improvements in pain, fatigue, sleep/rest activity, depression and morning stiffness. The combination of fluoxetine with melatonin caused even greater significant reductions in both anxiety and depressive symptoms, with reduction of fatigue symptoms in addition to each of the symptoms improved by each drug given individually. A noteworthy point emerging from this study is that with all treatment modalities (with fluoxetine, with melatonin or combination of both) there was a significant reduction of pain symptoms in fibromyalgia patients, showing the efficacy of the SSRI antidepressant fluoxetine and of melatonin in fibromyalgic pain [111]. Additional clinical studies in fibromyalgia patients indicate that melatonin therapy will be effective in treating pain associated with fibromyalgia [112, 113]. 

### b Melatonin use in Irritable Bowel Syndrome

Another clinical condition in which melatonin has been found useful is irritable bowel syndrome (IBS). This condition is associated with abdominal pain, flatulence, constipation, diarrhoea and sleep disturbances. There are two randomized placebo-controlled clinical trials administering 3 mg/day of oral melatonin. In one study conducted in 40 patients, melatonin or placebo was given for two weeks [114]. Reductions in abdominal and rectal pain were noted. In another study in 24 women with IBS employing a crossover design, patients received melatonin or placebo for 8 weeks followed by a 4-week washout period, after which they received the other treatment [115]. A reduction in pain symptoms with improvements in the IBS was noted in all 24 patients. 

### c Melatonin use in Migraine

In migraine, a clinical condition associated with attacks of severe headache and sleep-disturbances, an association between nocturnal melatonin secretion and symptoms of migraine has been studied. Significantly lower urinary melatonin levels were found in ten migraine patients when compared to nine healthy controls [116]. Similarly, in a study in a large sample of 146 migraine sufferers with additional pain syndromes, significantly lower urinary 6-sulphatoxy-melatonin was found in this group when compared to controls or migraine patients without additional pain syndromes [117]. In a study of migraine sufferers with disturbances in melatonin secretion (phase advance or phase delay), daily 5 hr infusions of 4 µg melatonin/hr relieved morning headache in four patients after the first infusion and two others obtained relief from morning headache after the third infusion thereby showing a beneficial effect of melatonin in relieving the pain associated with migraine [118]. In a recent study, the melatonin agonist agomelatine has been used successfully for treating patients with migraine attacks. Agomelatine administered to patients suffering from migraine attacks (in doses of 25 mg/day for the duration of six months) decreased both the frequency and duration of migraine attacks and thus reduced the intensity of pain in migraine patients. Moreover, it also reduced significantly the severity of depression and normalized sleep disturbances [119]. This study, the first of its kind, proves the beneficial effect of this melatonergic antidepressant in treating migraine attacks.

### d Melatonin and Cluster Headache

Melatonin is also involved in the pathogenesis of cluster headaches since its circadian rhythm is disrupted in this disorder [120]. Decrease in nocturnal melatonin secretion with loss of melatonin rhythm has also been identified in cluster headache patients [121]. Based on these studies, melatonin was tried in the treatment of cluster headache patients in a double blind, placebo controlled trial, in which melatonin caused significant decrease of cluster headache attacks in the melatonin treated group when compared to the control group [122]. In another study also melatonin in 9 mg dose not only prevented the nocturnal cluster headaches but also prevented the daytime attacks as well [123]. These studies support the involvement of melatonin in the pathophysiology of cluster headaches. Pineal cysts which account for 1.3% to 2.6% of brain MRIs have been associated commonly with headache patients. In a study by Peres *et al., *(2005) [124], pineal cysts were found in 5 headache patients (4 women and 1 man), two patients had migraine without aura, one had migraine with aura, one chronic migraine, and one hemicrania continua. Abnormal melatonin secretion and the presence of pineal cysts were found to be associated with headache in this group of patients. A number of mechanisms like free radical scavenging, anti-inflammatory effects, inhibition of nitric oxide synthase activity, inhibition of dopamine release, GABA potentiation, and neurovascular regulation, have all been proposed for the beneficial effects in treatment of cluster headaches and migraine attacks [124]. In an experimental animal model of headache induced by capsaicin, c-fos response expression in trigeminal nucleus caudalis (TNC), was studied in control animals, pinealectomized animals, animal received capsaicin and melatonin. In this study pinealectomized animals receiving capsaicin presented the highest number of c-fos positive cells in the trigeminal nucleus caudalis, whereas animals injected with capsaicin and melatonin had only and lower expression of the c-fos response as has been found in the control vehicle treated group, showing melatonin’s beneficial action in this experimental model [125].

## 10 MELATONIN’S ANTINOCICEPTIVE EFFECTS: POSSIBLE MECHANISMS OF ACTION

Early studies on melatonin’s antinociceptive actions have shown that both opiate and BZD pathways are involved. The relationship of melatonin with the opioidergic system is complex. Recent studies suggest that melatonin can exert its antinociceptive effects by acting indirectly through a number of neurotransmitter systems and their related receptor sites including benzodiazepinergic (BZD receptor), opioidergic (δ/κ/μ receptors), sigma system (sigma receptor), serotonergic (5HT_2A_ receptor), dopaminergic (D_2_ receptor), adrenergic (α_2_ receptor), glutamatergic (NMDA receptor), NO-cyclic GMP-PKG signalling pathway, and, directly, through melatoninergic MT_1_/MT_2_ receptors [126]. Evidence for these interactions includes the reversal of melatonergic antinociceptive effects by the BZD antagonist flumazenil [55], (δ/κ/µ opioid and sigma receptor antagonist naloxone [60, 62, 80], 5HT_2A_ receptor antagonist ketanserin [127], D_2_ receptor antagonist sulpiride [127], the α_2_-adrenoceptor antagonist yohimbine [127], and the effects of the NOS modulator L-arginine [62]. Evidence of direct melatoninergic antinociception involves the MT_1_/MT_2_ antagonist luzindole [76] and the MT_2_ antagonist 4-P-PDOT [69, 81]. Although modulation of these pathways play a role in melatonin’s antinociceptive effects, the interaction of melatonin with both central and peripheral MT_1_/MT_2_ melatonin receptors seems crucial for melatonin’s antinociceptive effects [59]. 

NMDA receptors in the spinal cord have been shown to be important in the transmission of pain sensation and potentiate spinal cord nociceptive synaptic transmission, an effect known as “wind-up,” in which there is a repetitive increase in the intensity of C fiber stimulation [2, 128]. In a paradigm stimulating C fibers and inducing this “windup effect,” melatonin‘s antinociceptive effect was evaluated. Different doses of melatonin (1.25, 2.5, 5.0, 10.0 mg/kg) produced dose dependent decreases of “spinal-wind up,” resulting in a complete suppression of windup at higher doses. 

Melatonin’s influence on spinal wind-up is attributed to its action on NMDA receptor-dependent intracellular NO generating pathways [129]. Nitric oxide has diverse roles in nociceptive transmission. It interacts with NMDA receptors and COX and induces a hyperalgesic effect, but by acting through cGMP-PKG-ATP sensitive K^+^ channels, NO also exerts an antinociceptive effect [13, 130]. Thus, melatonin interacts with a variety of systems, including benzodiazepine, opioidergic, serotonergic, dopaminergic, adrenergic, glutamatergic, the NO-cyclic GMP-PKG signalling pathway, and through diverse receptors including BZD, (δ/κ/μ, sigma, 5-HT_2A_, D_2_, α_2_, NMDA, NO and MT_1_/MT_2_ receptors.

## 11 ROLE OF MELATONIN RECEPTORS IN ANTINOCICEPTION

Melatonin produces antinociception by inhibition of α_1_-adrenergic, α_2_-adrenergic, muscarinic and nicotinic receptors in the spinal cord. One possible mechanism of action is suggested to be the activation of the cGMP system. Hence it is possible that intrathecal melatonin by increasing cGMP may recapitulate the stimulation of α_1_-adrenergic, α_2_-adrenergic, muscarinic, and nicotinic receptors, thereby producing antinociception [83]. Melatonin’s exertion of direct antinociceptive effects through activation of MT_1_ and MT_2_ melatonin receptors has been demonstrated in a number of experimental studies on animals. This has been made possible by use of the common melatonin receptor (i.e., MT_1_/MT_2_) antagonist luzindole, and specific MT_2_ receptor antagonists like 4-P-PDOT or K185, (N-butanoyl-2-(5,6,7-trihydro-11-methoxybenzo[3,4]cyclohept[2,1a]indole-13-ylethanamine). The antinociceptive effects of melatonin in neuropathic pain models and the localization of MT_1_/MT_2_ melatonin receptors in the thalamus, hypothalamus, dorsal horn of the spinal cord, spinal trigeminal tract and trigeminal nucleus suggest that antinociceptive actions of melatonin are mediated through melatonergic receptors. Not only melatonin’s antinociceptive effect but also the antinociceptive effect of the melatonin agonist 2-bromo-melatonin was blocked by intrathecal administration of luzindole [131-133]. Similarly the antinociceptive effect of intrathecal injection of 6-chloromelatonin was blocked by intrathecal administration of the melatonin MT_2_ receptor blocking agent, 4-P-PDOT [74]. The distribution of MT_1_ and MT_2_ melatonin receptors in lamina I-V and X of the spinal cord, and their possible role in the mediation of antinociceptive actions of melatonin suggests that melatonin receptors do play a major role in the modulation of pain transmission pathways in the spinal cord as well as in the supraspinal region [104]. A schematic diagram showing the possible mechanisms of antinociceptive action is presented in Fig. (**2**).

Melatonin has been tried as premedication for surgical operating conditions like cataract surgery, and also during tourniquet-related pain and in both these conditions reduced anxiety, improved preoperative analgesia, decreased tourniquet-related pain and enhanced intra-operative and post-operative analgesia [134, 135].

## CONCLUSION

Antinociceptive actions of melatonin have been well demonstrated in a number of animal studies by using different types of models including acute pain, inflammatory pain and neuropathic pain. While elucidating the mechanism of antinociceptive effects of melatonin, it has been noted that melatonin interacts with a number receptor sites including opioidergic, benzodiazepinergic, muscarinic, nicotinic, serotonergic, α_1_-adrenergic, α_2_-adrenergic and most importantly MT_1_/MT_2_ melatonergic receptors present in the dorsal horn of the spinal cord as well in the central nervous system. Not only melatonin but melatonin agonists such as 2-bromomelatonin, 6-chloromelatonin and certain other pyrrolol indole derivatives of melatonin also exert significant anti-inflammatory and analgesic activity. In this context it is suggested that the novel melatonergic antidepressant agomelatine may have a promising role in treating neuropathic pain associated with inflammation and nerve injury. In clinical disorders associated with chronic pain, e.g fibromyalgia, depression, migraine, and IBS and cluster headaches, melatonin’s use has been found beneficial in alleviating pain associated with these disorders. In acute surgical conditions, melatonin’s use reduced anxiety and enhanced both pre-operative and post-operative analgesia. Melatonin has been shown to be beneficial. Perhaps melatonin agonists with longer half-lives than melatonin (20-30 minutes) such as agomelatine (2 hours) or even ramelteon (1-2 hours) will also find clinical utility in the effective management and treatment of chronic pain and its associated clinical disorders.

## Figures and Tables

**Fig. (2):**
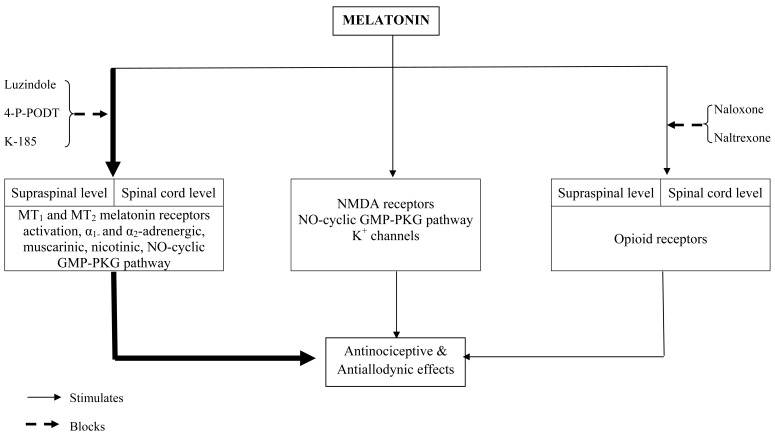
Schematic Diagram Showing Melatonin’s Antinociceptive Actions.

**Table 1.** Melatonin's Antinociceptive Actions - Experimental Animal Studies

Animal Model Used	Melatonin or Its Agonist Dose & Route	Effect of Melatonin	Blocked by	Type of Receptors Involved	References
Hot-plate	30 mg/kg i.p (melatonin)	Antinociception	Naloxone	Opioid	[26]
Hot-plate	20-40 mg/kg i.p (melatonin)	Antinociception	Naloxone & flumazenil	Opioid & BZD	[60]
Carrageenan-induced paw inflammation	5 & 10 mg i.p (melatonin)	Reduction of paw inflammation & antinociception	-	-	[66]
LPS model	5 & 10 mg i.p (melatonin)	Antinociception	-	-	[64]
Hot-water tail flick test	30, 60 or 120 mg/kg i.p (melatonin)	Antinociception	Naloxone	Opioid	[62]
Electrical stimulation of tail	0.5 & 1.0 mg i.p (melatonin)	Antinociception	-	-	[65]
Carrageenan-induced paw inflammation	5 & 10 mg i.p 0.25, 0.5, 1.0 mg i.c.v (melatonin)	Reduction of inflammation & antinociception	-	-	[74]
Paw-withdrawal threshold	70 mg/kg i.v cumulative dose (210 mg/kg) (melatonin)	Antinociception	Naloxone or luzindole	Opioidergic and melatonergic	[75]
Tail-clamping response	38 mg/kg (35-41 mg/kg) (bromomelatonin)	Antinociception	-	-	[76]
Capsaicin-induced hyperalgesia	Melatonin, 6-chloromelatonin	Antinociception	4-P-PDOT	MT_2_ melatonin	[69]
Ligation of sciatic nerve (neuropathic pain)	120 mg/kg i.v 0.1 nmol i.c.v (melatonin)	Antinociception	Naloxone	Opioid peptides & L-Arginine-NO-pathway	[80]
Ligation of spinal nerves (neuropathic pain)	37.5-300 mg oral 3-100 µg intrathecal (melatonin)	Antinociception	Luzindole both oral and intrathecal & 4-P-PDOT	MT_1_ and MT_2_	[81]
Formalin injection model	150 mg/kg oral (melatonin)	Antinociception	4-P-PDOT	MT_2_	[132]
Hot-plate latency test	4 mg/kg s.c (melatonin) and 5.16, 5.13, 6.88 & 5.40 mg/kg s.c (melatonin analog, compounds 3, 5, 9a, & 12)	Antinociception	-	-	[67]
Inflammatory pain model	150-600 µg/paw (melatonin)	Antinociception			[71]
Post-inflammatory visceral hyperalgesia to colorectal distension	60 mg/kg (melatonin)	Antinociception	Naltrexone or luzindole	Opioid or melatonin	[133]
Inflammatory type of pain	25-100 mg/kg (melatonin)	Antinociception	-	-	[42]
Inflammatory pain	3, 10 and 30 µg intrathecal (melatonin)	Antinociception	Prazosin, yohimbine, atropine, mecamylamine	α_1_- & α_2_-adrenergic, nicotinic and muscarinic	[73]
